# Community spread and late season increased incidence of oseltamivir‐resistant influenza A(H1N1) viruses in Norway 2016

**DOI:** 10.1111/irv.12637

**Published:** 2019-03-04

**Authors:** Karoline Bragstad, Olav Hungnes, Irene Litleskare, Hans Christian Nyrerød, Dagny H. Dorenberg, Siri H. Hauge

**Affiliations:** ^1^ Department of Influenza Norwegian Institute of Public Health Oslo Norway; ^2^ Department of Drug Statistics Norwegian Institute of Public Health Oslo Norway; ^3^ Department of Anesthesiology Oslo University Hospital, Rikshospitalet Oslo Norway

**Keywords:** antiviral resistance, H275Y, influenza, surveillance

## Abstract

**Background:**

Antiviral resistance in Norwegian influenza viruses is rare. Only one A(H1N1)pdm09 virus from May 2015 had been found resistant to oseltamivir since the introduction of these viruses in 2009.

**Objectives:**

Surveillance of antiviral resistance is part of the Norwegian surveillance system, to rapidly detect the development of antiviral‐resistant viruses and spread in the community. We describe the spread of oseltamivir‐resistant A(H1N1)pdm09 viruses in Norway in the 2016‐17 season, found as part of the routine surveillance.

**Methods:**

Influenza H1N1 viruses were analysed for antiviral resistance by pyrosequencing, neuraminidase susceptibility assay and by Sanger sequencing of the HA and NA genes.

**Results:**

During the 2015‐16 influenza season, 3% of all A(H1N1)pdm09 viruses screened for resistance in Norway were resistant to oseltamivir, possessing the H275Y substitution in the neuraminidase protein. In comparison, the overall frequency in Europe was 0.87%. Out of these, 37% (n = 10) were reported from Norway. Most cases in Norway were not related to antiviral treatment, and the cases were from several different locations of southern Norway. Genetic analysis revealed that resistant virus emerged independently on several occasions and that there was some spread of oseltamivir‐resistant influenza A(H1N1)6B.1 viruses in the community, characterised by a N370S substitution in the haemagglutinin and T48I in the neuraminidase.

**Conclusions:**

Our findings emphasise the importance of antiviral resistance surveillance in the community, not only in immunocompromised patients or other patients undergoing antiviral treatment.

## 1 INTRODUCTION

The National Influenza Centre for WHO in Norway (NIC Norway) is the only institution in Norway performing antiviral resistance testing and surveillance. The national surveillance system for influenza comprises a network of volunteer sentinel physicians and medical microbiology laboratories which report weekly the number of positives and the number of specimens tested. Furthermore, they send positive specimens to the NIC for further characterisation. A selection of surveillance samples are screened for antiviral resistance by real‐time PCR and sequencing and/or susceptibility to antivirals in neuraminidase inhibition assay. Norway runs a well‐functioning influenza surveillance programme that benefits from comprehensive diagnostic testing for influenza at the regional laboratories with over 110 000 samples tested during the 2015/16 season, nearly 15 000 samples of these were found influenza positive. Approximately 3000 of these samples are shipped to the NIC for further characterisation and enrolment in the global surveillance. A(H1N1)pdm09 viruses (further referred to as H1 or H1N1), subclade 6b.1, dominated the 2015/16 season.

The most commonly used neuraminidase inhibitors (NI)—oseltamivir (Tamiflu^®^) and zanamivir (Relenza^®^)—are authorised for use in Norway, but only oseltamivir is available on the market from 2016. The use of antivirals differs globally with the United States and Japan as the major consumers with eight million NI prescriptions annually in Japan.^1^ Antivirals are not widely used in Norway and mainly recommended for at‐risk and severely ill patients, with approximately one course sold pr 1000 inhabitants in 2016 (Table 1).

**Table 1:** Oseltamivir‐resistant cases in Norway in the 2015‐16 season

Case	Isolate	County	Region	Sampling date	Age	Sex	Status	Antiv. treat.^a^	Outcome	IC50 Oselt.	IC50 Zanam.	Sample	Oselt. Res. a	Zanam. Res. b	Res. Mut.	Acc_no_HA	Acc_no_NA
1	A/Norway/2914/2015	Aust‐Agder	South	14.12.2015	4	M	O	N	U	723	3.5	Throat	HRI	NI	275Y	EPI695299	EPI700046
2	A/Norway/411/2016	Østfold	East	19.01.2016	30	M	O	U	U	ND	ND	Nasopharynx	AAHRI	AANI	275Y	EPI759009	EPI759182
3	A/Norway/541/2016	Hordaland	West	25.01.2016	50	F	O	U	U	ND	ND	Nasopharynx	AAHRI	AANI	275Y	EPI759014	EPI759183
4	A/Norway/1476/2016	Hedmark	East	02.03.2016	57	F	O	U	U	301	0.6	Nasopharynx	HRI	NI	275Y	EPI759038	EPI759188
5	A/Norway/1828/2016	Buskerud	East	04.03.2016	66	F	H	N		389	1.0	U	HRI	NI	275Y	EPI759045	EPI759193
6	A/Norway/1759‐2/2016	Nord‐Trøndelag	Middle	09.03.2016	57	M	H	Y	I + D	ND	ND	Bronchial	AAHRI	AANI	275Y	EPI759044	EPI759191
6	A/Norway/1759‐3/2016	Nord‐Trøndelag	Middle	09.03.2016	57	M	H	Y	I + D	ND	ND	Nasopharynx	AAHRI	AANI	275HY (48%)	No sequence	EPI759192
7	A/Norway/2036/2016	Hedmark	East	10.03.2016	53	F	H	Y		510	1.9	U	HRI	NI	275Y	EPI759048	EPI759194
8	A/Norway/2114/2016	Buskerud	East	18.03.2016	51	M	O	N	U			U	AAHRI	AANI	275Y	EPI759049	EPI759195
9	A/Norway/2298/2016	Buskerud	East	21.03.2016	78	M	H	N		246	0.7	Nasopharynx	HRI	NI	275Y	EPI759051	EPI759196
10	A/Norway/2404/2016	Vestfold	East	21.03.2016	51	F	O	N	U	ND	ND	Nasopharynx	AAHRI	AANI	275Y	EPI759053	EPI759197

AAHRI, amino acid substitution previously associated with highly reduced inhibition; AANI, amino acid substitution previously associated with normal inhibition; D, dead; F, female; H, histidine; H, hospitalised; HRI, highly reduced inhibition (>100‐fold increase in IC50); I, intensive care; M, male; N, no; ND, not done; NI, normal inhibition (<10‐fold increase in IC50); O, outpatient; U, unknown; Y, tyrosine; Y, yes.

a Antiviral drugs are very seldom used in Norway. Antiviral treatment (oseltamivir) is mainly given to patients with severe respiratory disease in critical care.

b Wild‐type IC50 median for oseltamivir‐sensitive H1N1 virus 0.9, zanamivir‐sensitive virus 0.5.

Resistance against the antiviral drugs occurs through mutations in the viral genome. Resistance to NI is caused by single point mutations in the neuraminidase (NA) gene. Substitutions in several codons have been identified, in vitro, to cause different levels of resistance against the different drugs.^2^ The H275Y mutation (N1 numbering) reduces susceptibility of H1N1 influenza virus to oseltamivir by more than 400‐fold and also reduces susceptibility to peramivir, but does not cause resistance to zanamivir in vitro.^3^ Antiviral‐resistant viruses do emerge sporadically. It was initially believed that antiviral resistance mutations would be generally unfavourable for virus infectivity and transmission. However, in 2007, Norway reported unprecedentedly high proportions of oseltamivir resistance in former seasonal H1N1 viruses, due to the H275Y substitution in the NA gene. Obviously, these viruses had not suffered a loss in fitness and appeared in many other countries at the same time. Within 1 year, the resistant virus had become the predominant H1N1 strain globally.^4^, ^5^, ^6^ The first case of resistant pandemic H1N1 virus was reported early in the 2009 pandemic in Denmark.^7^, ^8^, ^9^ Local clusters/incidences of oseltamivir‐resistant H1N1 viruses have been reported from Hokkaido, Japan,^10^ Pennsylvania, United States and Australia.^11^, ^12^ It has been shown that H1N1 viruses possessing the NA H275Y amino acid substitution are able to replicate and transmit as efficiently as normal wild‐type viruses, provided that mutations are present in other positions that make up for the prior disadvantage of the H275Y substitution.^13^, ^14^

Here, we report increased incidences of H1N1 viruses with highly reduced inhibition (HRI) by oseltamivir, possessing the H275Y NA substitution, during the 2015/16 influenza season in Norway.

## 2 METHODS

Influenza viruses were obtained as part of the national surveillance system for influenza in Norway. Medical microbiology laboratories (non‐sentinel), representing all 19 counties, submit a selection of samples PCR positive for each influenza virus type to the NIC every week. General practitioners enrolled in the national influenza surveillance programme (sentinel n = ~70) collect samples from patients with influenza‐like illness and send them for diagnostic testing at the NIC. The NIC receives a nearly even distribution of samples from outpatients (both non‐sentinel and sentinel) and hospitalised patients (non‐sentinel).

Samples were tested for antiviral resistance both genetically and phenotypically. Nucleic acid (200 µL) was extracted from clinical samples using the MagNA Pure 96 DNA and Viral RNA Small Volume Kit (Roche diagnostics, Basel, Switzerland), typed^4^ and H1 subtyped^15^ by real‐time RT‐PCR on RotorGene cycler system (Corbett/QIAGEN, Hilden, Germany). H1N1 viruses were screened for the H275Y mutation by pyrosequencing^16^ on the PyroMark Q96 ID (QIAGEN) with primers: sw‐N1‐F780B gggaaagatagtcaaatcagtcga, sw‐N1‐R1273E (5′‐biotin) CAACCCAGAAGCAAGGTCTTAT, sw‐N1‐F804Bseq AATGAATGCMCCTAATT. The NA gene was also fully or partially sequenced by Sanger sequencing on Applied Biosystems 3500xL Genetic Analyzer (Thermo Fisher Scientific, Waltham, MA, USA) (PCR and sequencing primers available upon request). Viruses for phenotypic resistance analysis were isolated/propagated in MDCK cells, one to two passages.^17^ The susceptibility of virus isolates to oseltamivir and zanamivir was measured by a neuraminidase inhibition assay, applying 20‐(4‐methylumbelliferyl)‐a‐D‐N‐acetylneuraminic acid (MUNANA) substrate^18^ to determine the concentration of drug that inhibits the neuraminidase activity by 50% (IC50).^19^ Whereas functional drug susceptibility was ascertained on MDCK grown virus, all genotypic testing was performed on original clinical material.

H1 viruses were characterised as oseltamivir‐resistant by the measurable (ie >10% of NA‐encoding RNA) presence of the H275Y substitution in the NA protein and/or with elevated neuraminidase inhibitory IC50 values compared to the mean IC50 of susceptible samples in current and previous seasons. An isolate with a 10‐ to 100‐fold increase in IC50 was classified as having “reduced inhibition” (RI) and with more than 100‐fold increase in IC50 as “highly reduced inhibition” (HRI). Viruses that could not be propagated for NA susceptibility studies were characterised as “amino acid substitution previously associated with highly reduced inhibition” (AAHRI) by the presence of the H275Y substitution. Nucleotide sequences were further analysed by BioNumerics (Applied Maths, Sint‐Martens‐Latem, Belgium), BioEdit^20^ and MEGA 6 software.^21^ Phylogenetic trees were constructed with the neighbor‐joining method, using Kimura 2‐parameter pairwise distances. Nucleotide cluster analysis was performed with maximum parsimony trees. Sequences were submitted timely to the EpiFlu database hosted by the Global Initiative on Sharing All Influenza Data (GISAID). The accession numbers of resistant Norwegian viruses are given in Table 1. The amino acids positions are described using N1 numbering.

Antiviral sales figures were extracted from the Norwegian Drug Wholesales Statistics and the Norwegian Prescription Database (NorPD). The wholesales statistics contain complete data on all medicines sold, as packages sold and number of defined daily doses (DDD), from the wholesalers to Norwegian pharmacies, hospitals and nursing homes. We calculated courses sold per. 1000 inhabitants based on population data from Statistics Norway. The prescription database receives reports from all pharmacies in Norway on prescriptions filled by outpatients registered on their unique personal identification number.

## 3 RESULTS

**Figure 1:**
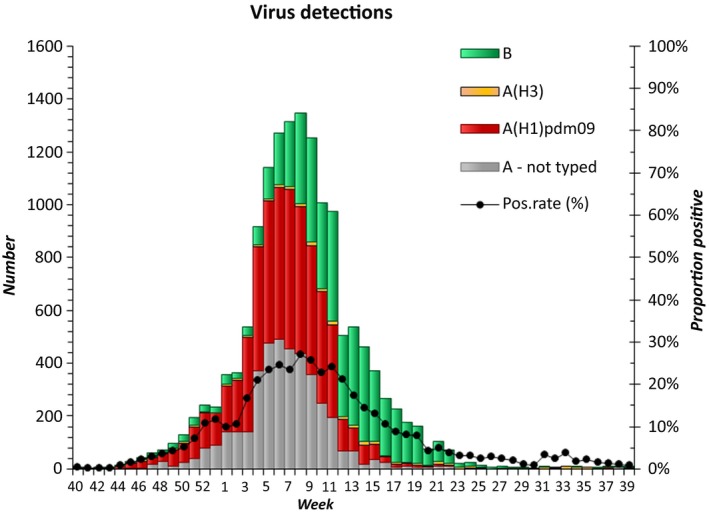
Laboratory detections, Norway, 2015‐16. Weekly numbers of the different influenza viruses are displayed as stacked bars, and influenza virus positivity rates of all laboratory testing are shown as line graphs

Influenza A viruses accounted for 69% of the influenza virus detections in the 2015‐16 season in Norway. As in most European countries that season, H1N1 was accounted for more than 90% of sentinel influenza A viruses in Norway. Activity peaked at intermediate levels in late February, weeks 5 through 8. From week 12 onwards, influenza B viruses of the Victoria/2/1987 lineage predominated until settling at low levels in early June (Figure 1). The H1N1 viruses mostly belonged to the 6B.1 clade.^22^ In total, 2580 samples were collected for further analysis this season (276 H3N2, 1310 H1N1 and 994 influenza B). Out of the H1N1 viruses received, 571 were from hospitalised patients and 734 were from outpatients (out of these, 75 were from the sentinel system of GPs). We performed neuraminidase susceptibility testing on 326/1310 (28%) of the H1N1 viruses, 264 of the H3N2 viruses (24%) and 69 of the influenza B viruses (7%) that were received at the NIC in the 2015/16 season. The neuraminidase gene was sequenced either by pyro‐ or Sanger sequencing in 301 samples.

**Figure 2:**
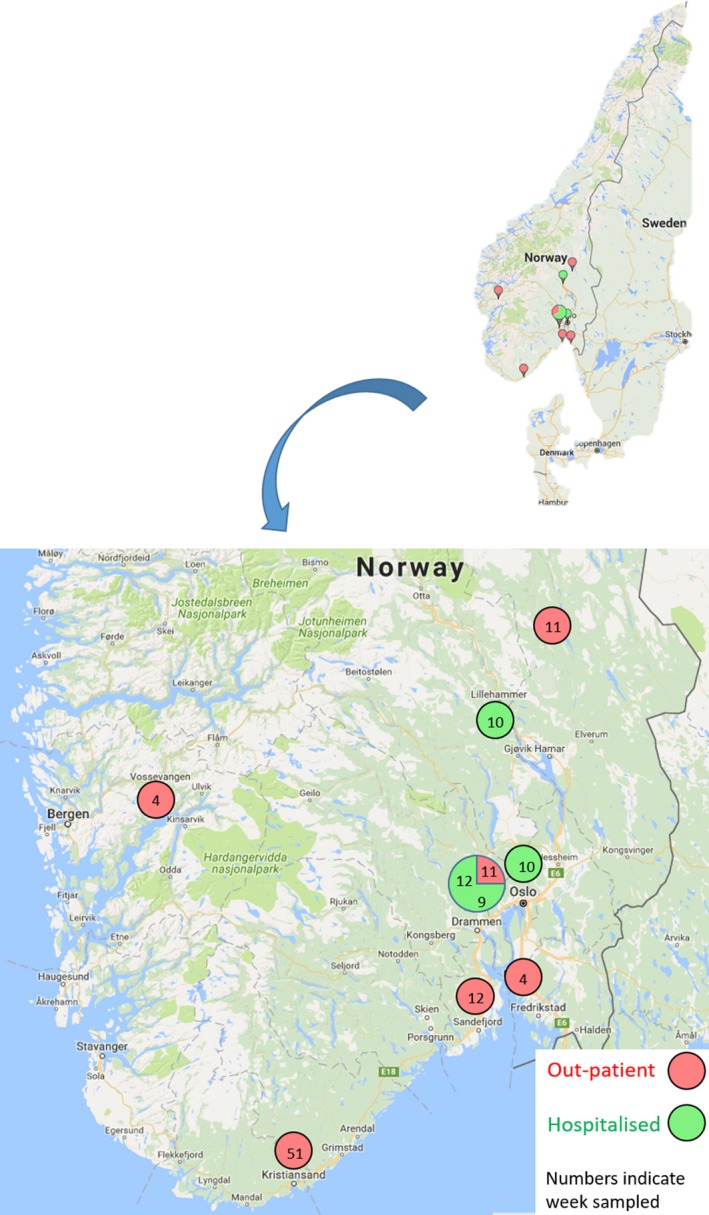
Geographic localisation of oseltamivir‐resistant cases in Norway in the 2015/16 season. Oseltamivir‐resistant viruses from outpatient (red) and hospitalised persons (green) at different time periods (wk) and locations in the southern part of Norway in the 2015‐16 season. Numbers indicate week of sampling

Since the H1N1 emergence during the 2009 pandemic and until the 2015/16 season, only one Norwegian virus, (A/Norway/2227/2015 H1N1pdm09), collected in May 2015, had been found resistant to oseltamivir. The virus, possessing the H275Y substitution, was from an outpatient from south‐east of Norway, with no history of antiviral treatment or travel abroad. The first resistant H1N1 virus in the 2015/16 influenza season (A/Norway/2914/2015) was detected in December 2015 (Table 1). The sample was collected as part of the surveillance system and was from an outpatient child, with no exposure to antiviral drugs or travel history. By the end of March 2016, the proportion of samples with antiviral resistance to oseltamivir (275Y mutation) had increased to 3% of the tested H1N1 samples. In total, 10 out of the 326 H1N1 samples tested were found to be oseltamivir‐resistant (Table 1), all belonging to the HA genetic clade 6B.1. Six of these ten resistant cases were sampled from outpatients and four cases were from hospitalised patients. Seven out of these ten cases were sampled in March 2016. The six resistant samples from outpatients were from different parts of southern Norway (Figure 2) and were detected from week 51 in 2015 through week 12 in 2016. Three of the outpatients were known not to have been treated with antivirals. Two of the four hospitalised cases had received antiviral treatment. Hospitalised resistant cases were, as the outpatient samples, from different parts of southern Norway, but all were sampled in March 2016 (Figure 2).

**Figure 3:**
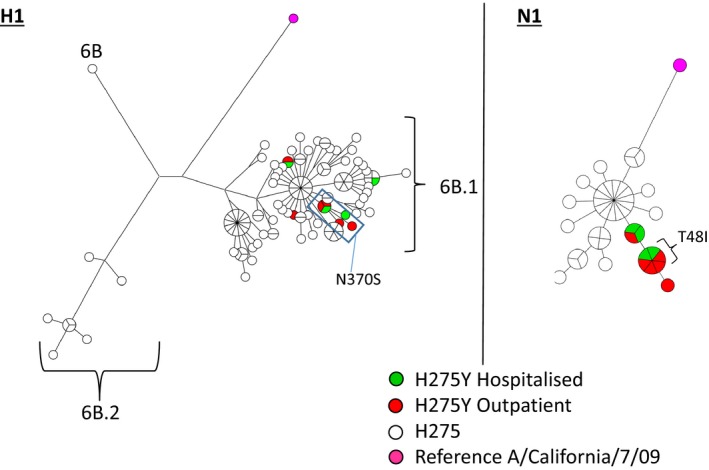
HA and NA gene cluster analysis of H1N1 virus in the 2015/16 season in Norway. HA and NA genetic diversity (nucleotide) of oseltamivir‐resistant Norwegian viruses from outpatients (red), hospitalised (green) patients and non‐resistant cases (open circles) with reference virus A/California/7/09(H1N1)pdm09. Genetic groups are indicated in the H1 cluster, and key amino acid substitutions defining the community‐spread oseltamivir‐resistant cluster are given. Cluster analysis was constructed with BioNumerics, and basic maximum parsimony tree was applied as network creation method

**Figure 4:**
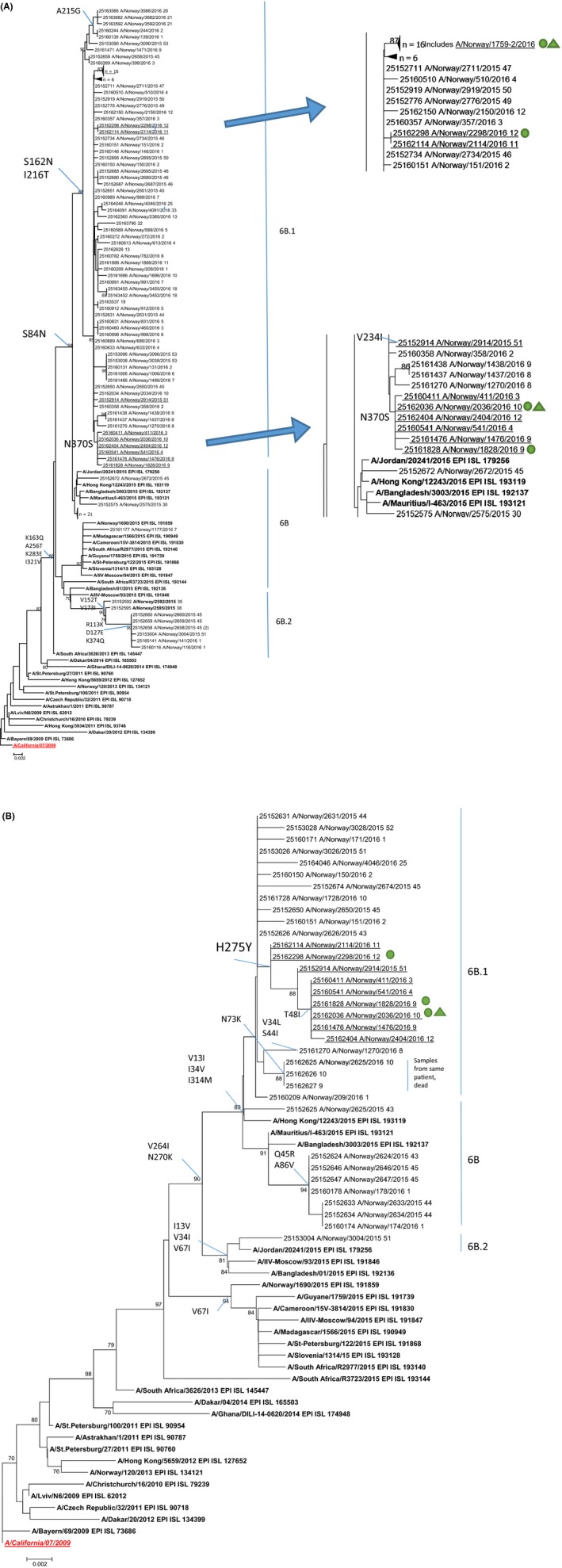
Phylogenetic reconstruction of Norwegian 2015‐16 A/H1N1pdm09 HA (A) and NA (B) genes. Reference viruses are in bold, vaccine strain 2015‐16 is marked in red bold italic and root is underlined. Aligned partial HA gene sequences (1010 bases) (A) and NA gene sequences (945 bases) (B) were subjected to phylogenetic analysis using neighbor‐joining of Kimura‐corrected genetic distances. Bootstrap values above 70% out of 500 resamplings are shown. Norwegian viruses from this season are named as “local ID_Isolate name_week.” Key amino acid differences to the reference A/California/07/2009 are indicated on key branch nodes. Oseltamivir‐resistant viruses from Norway in the 2015‐16 season are underlined. 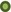, Hospitalised; 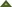, Treated with oseltamivir. Genetic H1N1 groups are indicated on the side of the tree

Six resistant viruses from different geographic regions, both from hospitalised and outpatients spanning from week 3 through week 12 of 2016, clustered together phylogenetically in both the HA and NA genes (Figures 3 and 4) and were closely related to the first resistant case from December. This cluster of viruses was characterised by amino acid substitution N370S in the HA stalk region and a signature silent nucleotide mutation to guanine at position 711 in the HA gene. In NA, these viruses were characterised by the amino acid substitution T48I, not found in other resistant cases or other cases from Norway and very few globally; only one single sample in GISAID EpiFlu, besides the Norwegian strains” possessed the T48I substitution in NA, but this sample lacked the H275Y substitution. It cannot be excluded, however, that a less than 50% presence of the substitution may have been overlooked in sequencing. Therefore, similar viruses have not been reported from other countries, which could imply that the virus has not spread further. This T48I substitution was not found in the first case of resistance (A/Norway/2914/2015), but this case had the signature nucleotide mutations uracil at positions 39 and 573 in common with the six later cases.

The hospitalised patient with sample A/Norway/1759/2016 (Table 1) developed resistance during treatment with oseltamivir (Table 1) but died shortly after. The virus in the initial bronchoalveolar lavage (BAL) sample taken 1 March (A/Norway/1759‐1/2016) was oseltamivir sensitive, carrying histidine in position 275. A nasopharynx sample (A/Norway/1759‐3/2016) from 9 March, following oseltamivir treatment, indicated development of resistance by 48% Y in position 275. Virus in a BAL sample from the same patient (A/Norway/1759‐2/2016) taken the same day was resistant to oseltamivir possessing the H275Y substitution (70%).

Another hospitalised case with resistant virus (A/Norway/2298/2016) (Table 1), who was treated with oseltamivir, groups phylogenetically together with the resistant virus from an outpatient (A/Norway/2114/2016) in both HA and NA genes (Figure 2). These viruses share one signature nucleotide mutation uracil at position 451, not found in any other Norwegian H1 viruses. Both persons (age > 50) lived in the same neighbourhood. The outpatient was sampled 3 days before the hospitalised patient (Table 1).

The phenotypic resistance analysis of viruses that we were able to propagate supported the genetic analysis of oseltamivir‐resistant viruses, with a 1000‐fold increase in IC50 confirming highly reduced inhibition by oseltamivir and normal inhibition by zanamivir (Table 1).

Antiviral use in Norway is low with sales of one courses/1000 inhabitants during 2016 (Table 2). Much higher sales were recorded during the 2009 H1N1 pandemic (Table 2). Hospitals represent approximately 31% of dispensed doses while prescriptions stand for 48% and nursery homes, GPs or other institutions for 22% measured in DDDs (based on wholesale numbers from 2015). The consumption is higher in adults than in elderly and children.

**Table 2:** Sales of influenza antivirals, Norway, 2009‐2016

Year	Wholesales statistics^a^				Prescriptions			
	Number of courses sold			Total courses sold per 1000 inhabitants	Number of courses sold			Total courses sold per 1000 inhabitants
	Oseltamivir	Zanamivir	Total		Oseltamivir	Zanamivir	Total	
2009	811 971	76 215	888 186	185.07	345 015	2986	348 001	72.51
2010^b^	−406 549	−67 804	−474 353	−97.64	4676	46	4722	0.97
2011	6471	−38	6433	1.31	3205	50	3255	0.66
2012	3489	−87	3402	0.68	2234	38	2272	0.46
2013	10 377	61	10 438	2.07	4552	94	4646	0.92
2014	3131	21	3152	0.62	1259	21	1280	0.25
2015	3678	66	3744	0.72	1699	59	1758	0.34
2016	5459	15	5474	1.05	2562	27	2589	0.50

a Wholesales statistics represent sales from wholesalers to pharmacies, hospitals and nursing homes. Prescriptions: Sales from the Norwegian Prescription Database (NorPD) are included in the wholesales statistics.

b The negative numbers in 2010 are a result of return of unused medicines to the wholesales after stockpiling due to the 2009 pandemic. This means that at least 50% of the courses sold in 2009 were not used. It is not possible to separate the sales of new packages in 2010 from the returns from 2009.

## 4 DISCUSSION

An unusually high percentage of antiviral resistance to oseltamivir was detected in influenza H1N1 viruses from Norway during the 2015‐16 season. The ten H1N1pdm09 oseltamivir‐resistant cases (3%) included both outpatients and hospitalised patients. The genetic analysis suggests that resistant virus emerged independently on several occasions and that there was some spread of oseltamivir‐resistant influenza A(H1N1) 6B.1 viruses in the community. The first case was detected in December in an outpatient. During the season, six of the resistant viruses formed a genetic cluster with resistant viruses from different geographic locations in Norway, spanning from week 3 to week 12. Five of these cases had no antiviral treatment and four of the cases were outpatients. The single patient undergoing treatment was sampled second last of the samples in the cluster; thus, it is likely that the resistance mutation was present at infection and did not arise as a consequence of treatment of that patient. It is therefore most likely that the resistant viruses from this cluster were able to persist in the community at least from late January and until the very end of the season. The signature NA substitution at position 48 (T48I) in NA of this cluster of viruses is located in the unstructured linker region (residue 35‐82) which connects the membrane anchor to the catalytic neuraminidase domain (residues 83‐469).^23^ The unique substitution in HA, N370S, is located in the stalk region of HA. The effect on virus transmission or infectivity caused by these substitutions is unknown.

Two other resistant viruses clustered together in both the HA and NA genes. The two viruses were found in one hospitalised patient with oseltamivir treatment and one outpatient from the same neighbourhood. The outpatient was sampled 3 days before the hospitalised patient. It is therefore not unlikely that the hospitalised patient was infected with an already resistant virus before treated with oseltamivir.

The last case of resistance was a result of antiviral treatment and was genetically distinguishable from the first case and the two other clusters of resistant viruses that season.

All Norwegian H1N1 viruses characterised possessed the V241I and N369K substitutions in NA, including the resistant viruses. These mutations are known to increase replication and transmission fitness^14^ and were reported during a widespread cluster of H275Y mutant H1 viruses in Australia in 2011.^12^ All Norwegian H1N1 viruses had lost the same glycosylation site in NA as reported in the Hokkaido cluster, due to the substitution N386K.^10^ These three substitutions are now present in the vast majority of circulating H1N1 viruses.

The proportion of oseltamivir‐resistant viruses carrying the H275Y substitution was 3%, while the overall frequency in Europe the same season was 0.87% (27 viruses),^24^ with 10 out of these 27 resistant H1 cases being of Norwegian origin. The global overall frequency for viruses with reduced inhibition (RI) or highly reduced inhibition (HRI) by neuraminidase inhibition (NAI) assay was 0.5% in 2014/15^25^ and 1.8% in 2015/16.^26^ The consumption of influenza antivirals in Norway is generally very low (Table 2) and is not believed to have contributed to increased prevalence of resistance. Studies suggest that approximately 64% of oseltamivir‐resistant cases detected have arisen in immunosuppressed patients and occurred after oseltamivir treatment, with only 12% of resistance developing without drug use^27^, ^28^; however, antiviral resistance in untreated patients is largely understudied. Development of resistance to oseltamivir during treatment occurred more among seasonal influenza A(H1N1) virus infections (27%) compared with seasonal influenza A (H3N2) (3%) or B (0%) viruses.^29^ In Norway 2016, most (6 out of 10) oseltamivir‐resistant H1N1 cases were outpatients not likely related to antiviral drug treatment. Community cases will only sporadically be picked up if antiviral resistance testing is mainly conducted on immunocompromised patients or hospitalised patient on antiviral treatment. Most resistant cases during the 2015‐16 season in Norway occurred at the end of the season in March. By the end of March, very few H1 cases were detected and by week 12 influenza B dominated until season ended. The following season (2016‐17) was dominated by H3N2 viruses in Norway and Europe with very few H1N1 viruses circulating, and the 2017‐18 season was dominated by influenza B, also with few H1N1 viruses. The frequency of antiviral‐resistant H1N1 viruses in Europe increased from 0.4% in 2014/15 to 0.9% in 2015/16 to 1.9% both 2016/17 and 2017/18 (Flu News Europe). The overall prevalence of resistant influenza strains was between 0.3% and 0.9%.

Antiviral resistance testing on community viruses should be a priority, especially in the following seasons dominated by H1N1 viruses. As mentioned, there have previously been other incidences of resistant H1N1 community clusters as in Australia in 2011^12^ and in Hokkaido, Japan, in 2013‐14.^10^, ^30^ None of the clusters persisted until the subsequent season, as the mutant resistant viruses faded out in spring and other, imported, H1N1 viruses founded the next‐season circulating strains in autumn. Our results emphasise the importance of surveillance of antiviral resistance, not only in immunocompromised patients or during treatment; community surveillance is of equal or higher priority. It is worrying that we found this high frequency of resistance in Norway and this report will hopefully increase the awareness of resistant viruses circulating in the community. Many countries have to rely solely on oseltamivir as antiviral treatment; zanamivir is no longer available on the Norwegian market due to low sales, therefore options for alternative antivirals are needed.
